# *Tackle your Tics*: pilot findings of a brief, intensive group-based exposure therapy program for children with tic disorders

**DOI:** 10.1007/s00787-020-01532-5

**Published:** 2020-05-20

**Authors:** A. P. Heijerman-Holtgrefe, C. W. J. Verdellen, J. M. T. M. van de Griendt, L. P. L. Beljaars, K. J. Kan, D. Cath, P. J. Hoekstra, C. Huyser, E. M. W. J. Utens

**Affiliations:** 1 Dutch Tourette Association, Alkmaar, The Netherlands; 2 Dutch Knowledge Centre for Child and Adolescent Psychiatry, Utrecht, The Netherlands; 3 Amsterdam UMC University Medical Centers, Amsterdam, The Netherlands; 4 Parnassia Group/PsyQ, Nijmegen, The Netherlands; 5 TicXperts, Heteren, The Netherlands; 6 Parnassia Group, Den Haag, The Netherlands; 7 Research Institute of Child Development and Education, University of Amsterdam, Amsterdam, The Netherlands; 8 Department of Specialized Training, GGZ Drenthe, Assen, The Netherlands; 9 University of Groningen, University Medical Center Groningen, Groningen, The Netherlands; 10 Department of Child and Adolescent Psychiatry, University of Groningen, University Medical Center Groningen, Groningen, The Netherlands; 11 De Bascule, Academic Centre for Child and Adolescent Psychiatry, Amsterdam, The Netherlands; 12 Department of Child and Adolescent Psychiatry/Psychology, Erasmus MC, Sophia Children’s Hospital, Rotterdam, The Netherlands

**Keywords:** Tourette syndrome, Chronic tic disorder, Exposure and response prevention, Intensive behavioral treatment, Quality of life

## Abstract

Tourette syndrome (TS) and other chronic tic disorders (CTD) are prevalent neurodevelopmental disorders, which can have a huge burden on families and society. Behavioral treatment is a first-line intervention for tic disorders. Despite demonstrated efficacy, tic reduction and utilization rates of behavioral treatment remain relatively low. Patient associations point to an urgent need for easy-to-undergo treatments that focus both on tic reduction and improvement of quality of life. To enhance treatment outcome and overcome treatment barriers, this pilot study’s aim was to investigate the feasibility and preliminary results of a brief, intensive group-based treatment. *Tackle your Tics* is a 4-day intensive and comprehensive group-based program for children and adolescents (9–17 years) with a tic disorder, consisting of exposure and response prevention (ERP) treatment and additional supporting components, such as coping strategies, relaxing activities and parent support. Assessments were performed pre- and post-treatment and at 2 months follow-up, to test outcomes on tic severity and quality of life, and explore premonitory urges, emotional and behavioral functioning and treatment satisfaction (*N* = 14, of whom 13 completed the treatment). Parents and children rated this treatment positive on a treatment satisfaction questionnaire. On tic severity (Yale Global Tic Severity Scale) and quality of life (Gilles de la Tourette Syndrome Quality of Life Scale for children and adolescents), improvements between pre-treatment and follow-up were found. Intensive ERP in group format is promising as a feasible treatment to improve both tic severity as well as quality of life. Larger controlled trials are needed to establish its effectiveness.

## Introduction

Tourette syndrome (TS) and other Chronic tic disorders (CTD) are characterized by the presence of sudden motor movements and/or vocalizations that persist for more than a year, frequently associated with comorbid problems (e.g., hyperactivity, compulsions) [1]. Tic disorders are prevalent disorders (0.77–1% for TS [2, 3]) and can have a serious, long-lasting impact on quality of life [4–7], daily functioning [5], emotional/behavioral problems [8, 9] and school results [10]. Moreover, tic disorders can lead to stress of children and families, stigmatization [11], an elevated risk for suicide [12] and costs for society [4]. Tic severity alone does not define individual impairment or quality of life [7, 13]. Patient organizations emphasize the need for effective and available treatments, that not only focus on tic reduction but also on enhancing quality of life [14].

Behavioral treatment for tics is recommended as first-line intervention according to European clinical guidelines [15]. Several behavioral treatments for tics have been developed, specifically habit reversal (HR), Comprehensive Behavioral Intervention for Tics (CBIT) and exposure and response prevention (ERP). Research into behavioral treatment for tics report moderate-to-high effect sizes (0.57–1.5), but tic reductions remain relatively low (on average 30% on the Yale Global Tic Severity Scale, YGTSS) [16]. Research on ERP, however, is promising but limited. A comparative study of ERP versus HR showed that both treatments resulted in significant tic reduction with no difference between the two treatment conditions [17].

In addition, utilization rates for evidence-based behavioral therapies remain low [18, 19]. The lack of locally available trained therapists is a common treatment barrier. Families often have to travel far and as a consequence of low access, many children receive pharmacological treatment, despite a preference for behavioral treatments (C. Verdellen, J. Van de Griendt, N. Van de Berg, and S. Van Vugt; Dutch Tourette patients do not receive the treatment they prefer: data presented at the Tourettes association annual patients day 2014). Online behavioral treatment is increasingly being offered and investigated to solve the lack of specialized therapists, but offers few opportunities for peer and parent support and meetings.

Recently, two case studies in the USA and UK have suggested that brief, intensive forms of behavioral therapy for TS (which provided techniques as CBIT [20], ERP [21]) are as effective as weekly therapy sessions over a longer time frame. That is, in a clinical replication series of intensive CBIT (*N* = 5; [20]), an average decrease on the YGTSS total tic score of 28% was found in responders. Significant reductions of tic severity and improvements on daily functioning, quality of life, and emotional and behavioral difficulties were found after intensive ERP for children (provided in 2 consecutive days followed by weekly remote delivery by telemedicine) [21]. Besides, promising treatment outcomes have been found for an intensive ERP-based therapy for children with obsessive–compulsive disorder (OCD) [22–24]. Also, in other patient populations (e.g., adolescents with post-traumatic stress disorder, PTSD, receiving intensive ERP [25]), intensive forms of behavioral treatment have been successful. Moreover, research in anxiety disorders showed that treatment success may even be larger using intensive brief treatments compared to traditional approaches [26].

In addition, promising treatment outcomes have been found for an intensive outpatient group therapy (HRT) [27, 28] and in comprehensive CBIT-therapy that also focusses on secondary outcomes [7] in children with tic disorders. Benefits of group-based treatments may be peer support, reduced waiting lists, and increased cost-effectiveness. Nissen et al. compared combined HRT and ERP in a group setting versus in an individual setting and found no significant difference in total tic scores [40]. Himle examined group CBT for tic-related and non-tic-related OCD, showing that the treatment reduced OCD symptoms in both groups [29]. This indicates a possible improvement of comorbid symptoms in group therapy.

At present, there is a lack knowledge on the feasibility of an intensive form of behavioral treatment for tic disorders. Therefore, we developed a brief, intensive and comprehensive group-based ERP treatment program for children with tic disorders, *Tackle your Tics*. The aims of this pilot study were (1) to study the feasibility of *Tackle your Tics* and (2) to study treatment outcomes regarding tic severity (primary outcome), quality of life, premonitory urges and emotional/behavioral problems. We hypothesized that *Tackle your Tics* is a feasible treatment program for youths with TD. Furthermore, we expected to find indications of improvements in treatment outcomes. Importantly, we expect that *Tackle your Tics has* an additional surplus value in creating opportunities to train more behavioral therapists, in the specific tic-ERP techniques by experts in this field, since they can be co-therapists during this treatment. Increasing the number of trained therapists will lead to reduced treatment barriers.

## Methods

### Design

The present study is a pilot study, based on a small patient sample (*N* = 14) of children aged 9–14 years of age. Representatives of the Dutch national patient organization (among which author LPLB) were actively involved in this project, from start to finish, including developing the design of the study. This included continuous reviewing of the research process and the content of the project from the patients’ perspective by ‘experts by experience’, developing and performing workshops on coping strategies and parent support meetings during the treatment. Patients and their parents were recruited between June 2018 and December 2018 by the Dutch Tourette Association and the outpatient clinic De Bascule in Amsterdam. Tic severity and other inclusion and exclusion criteria were determined by an experienced child psychiatrist.

Inclusion criteria were: (a) youths aged 9–17 years, (b) diagnosed with TS or CTD, using diagnostic criteria of the Diagnostic and Statistical Manual of Mental Disorders, 5th edition [30], (c) with at least moderate tic severity as indicated by a YGTSS total score > 13 (or > 9 for children with motor or vocal tics only).

Exclusion criteria were: (a) behavioral treatment for tics in the past 12 months, (b) pharmacological treatment for tics that has not been stable the for the past 6 weeks or with planned changes during study participation, (c) poor mastery of the Dutch language, (d) IQ < 75, (e) serious physical disease, (f) substance abuse, (g) suicidality, (h) psychotic disorders, (i) severe autism spectrum disorders (ASD) or attention deficit hyperactivity disorder (ADHD) problems, which would hamper group functioning, (j) poor group functioning, as reported by child and/or parents during intake. Since TS is seldom seen without comorbidities [1], co-occurring ADHD, OCD, other anxiety disorders or mood disorders were included, unless the disorder required immediate treatment or change in current treatment.

Before the start of the therapy weeks, four co-therapists (psychologists with 2.5–5 years of experience) were trained by experts in behavioral therapy (ERP) for tic disorders. A patient advisory board, with parents and young adult patients, that gave feedback on the project, was installed during the study preparation phase and before treatment. Based on this feedback, some adaptations were applied to the program and data gathering. That is, we took into account their advice to guard against possible unrealistic expectations of the treatment outcomes (such as a complete disappearance of the tics) and possible misunderstanding of psycho-education, and to check the need for more care or support after the program, and the need for more tools to exercise at home. Therefore, we clarified the psycho-education for parents and added questions to the treatment satisfaction questionnaires. Also, the advisory board strongly recommended to build a positive and safe group atmosphere from the start, without children having to answer difficult questions about their problems. Therefore, we decided to collect the outcome measures at home, instead of during the treatment program at the outpatient clinic.

The children participated in one of two outpatient groups at De Bascule, Academic Centre for Child and Adolescent Psychiatry in Amsterdam, in September 2018 and February 2019, including, respectively, 6 and 8 children per group. Therapy sessions and psycho-education group meetings were performed by three highly experienced behavioral therapists, who are experts on tic disorders and ERP, assisted by three co-therapists, for training purposes. Coping workshops and parent meetings were developed and performed by experienced patient representatives of the Dutch Tourette Association.

### Intervention

*Tackle your Tics* is a group therapy program based on evidence-based ERP [31]. ERP aims to interrupt a postulated cycle of negative reinforcement between a premonitory urge and a subsequent tic by learning patients to tolerate premonitory urges while suppressing tics for prolonged time periods [17, 32]. To optimize exposure, urges are provoked, for instance by asking the patient to imagine situations with many tics, talking about tics and introducing urge-eliciting objects (e.g., exciting games). The therapist functions as a coach, encouraging the patient to improve his/her achievements. ERP usually consists of 12 weekly individual sessions (12 × 45 ERP-minutes = 540 min). In the *Tackle your Tics* program, ERP is provided in a brief, intensive format of 4 days: 3 consecutive days and 1 booster day after a week (4 × 135 ERP minutes = 540 min, see Table 1). The program was offered in groups of 6–8 children, to facilitate motivation and peer support. Apart from the overall group format, during *Tackle your Tics*, ERP exercises were trained in smaller subgroups of 2–3 children, in which children assisted each other (by timing, registering tics and encouraging). When needed, a child could train a specific exercise for a while individually with a therapist. On day 3, children went outside of the treatment center (e.g., riding a bike, being among other people, playing games) with the therapists to learn to generalize their newly learned skills. Also*,* several supporting and motivating activities were added to enhance motivation and fun, and reduce drop out.

**Table 1:** *Tackle your Tics* content

	Wednesday		Thursday	Friday	Follow-up day (after 1 week)	
09:30–10:00	Welcome and acquaintance game					
10:00–10:50	Psycho-education: drawing your tics and premonitory urges	10:00–12:00 Parent meeting 1: psycho-education and support	Group conversation: how did it go at home? Psycho-education: tic catching game (two children); education about tic triggers	Group conversation: how did it go at home? Psycho-education: Interviewing each other about tics and sensations (2 children); education about how to practice at home	Group conversation: how did it go at home? Psycho-education: education about easy and difficult moments, relapse prevention	10:00–12:00 Parent meeting 2: psycho-education and support
10:50–11:00	Short break		Short break	Short break	Short break	
11:00–12:15	Therapy session: individual training or 2/3 children assisting each other		Therapy session: individual training or 2/3 children assisting each other	Therapy session: individual training or 2/3 children assisting each other	Therapy session: individual training or 2/3 children assisting each other	
12:15–12:30	Relaxation exercises		Relaxation exercises	Relaxation exercises	Relaxation exercises	
12:30–13:00	Lunch break		Lunch break	Lunch break	Lunch break	
13:00–14:00	Workshop: learn to cope with tics and other symptoms in a positive way		Workshop: learn to cope with tics and other symptoms in a positive way	Workshop: learn to cope with tics and other symptoms in a positive way	Workshop: learn to cope with tics and other symptoms in a positive way	
14:00–14:30	Playtime		Playtime	Playtime	Playtime	
14:30- 15:30	Therapy session		Therapy session	Therapy session	Therapy session	
15:30–15:45	Short evaluation: what went well, what are your plans for tomorrow?		Short evaluation: what went well, what are your plans for tomorrow?	Short evaluation: what went well, what are you going to practice at home?	Short evaluation: what went well, what are your plans for the future?	
15:45–16:30	Feedback: therapist with (parents) and child		Feedback: therapist with (parents) and child	Feedback: therapist with (parents) and child	Feedback: therapist with (parents) and child	

#### Coping strategy workshops

Daily coping strategy workshops were given by trained young adult patients (experts-by-experience who are also educational professionals) from the national patient association. They taught the children how to cope with their symptoms in a positive ‘mind-set’. In accordance with a large European patient survey [14], this support did not focus on tics only but also on other symptoms, comorbidities and positive characteristics and strengths.

#### BT-Coach

*BT-Coach* is a mobile application that helps children to practice the ERP exercises, learned in the therapy sessions, in the absence of a therapist [33]. Through audio feedback, the app takes over the coaching role of the therapist during homework exercises. When a tic is expressed, the child registers this by pressing ‘Tap for a Tic’. BT-Coach stimulates the child to stop the next tic and work on new records. An improved version is currently being developed, in which data collection is optimized and calculations are possible. In this study, the BT-Coach was introduced during the first training days and sometimes used in the therapy sessions when children liked this. The app was used to motivate the children to continue with the exercises at home. On the third and fourth training day, the app was used as part of a relapse prevention plan (‘keep the tics away plan’). No data were collected for this study or in medical files.

#### Parent meetings

Parent meetings were in part attended by a therapist who explained the ERP treatment, discussed expectations and how parents could help their child gaining control over the tics during and after treatment. In addition, an experienced parent counsellor of the patient association offered parents the opportunity to exchange experiences in the parent group and find emotional support. At the end of each day, therapists had short individual meetings with the child and its parents to evaluate the day and ERP exercises, give advices on how to handle tics at home and answer possible questions.

#### Psycho-education

Children as well as their parents learned about premonitory sensations (‘tic alarms’), tic triggers, difficult moments and practicing at home.

#### Relaxation

The *Tackle your Tics* program contained several short relaxation trainings, focusing on breathing techniques, as well as playtime and fun activities.

### Measurements

Assessments were performed pre-treatment (T0, 1 week before treatment) and post-treatment (T1, 1 week after treatment: (that is: a week after the ‘booster’ day, to be able to measure the preceding week’s tic severity, after the booster session, by the YGTSS) and at the follow-up assessment (T2, 2 months after treatment).

Demographic variables/patient characteristics (gender, age, cultural background, parents’ educational level, comorbidities) were derived from medical files and a semi-structured interview (Anxiety Disorder Interview Schedule; ADIS, parent version) [34].

Feasibility was assessed by: (1) attendance/drop-out rates, (2) standardized treatment satisfaction forms (parent and child version, with a rating on five-point Likert scale, ranging from 1 = very negative/not helpful at all to 5 = very positive/helpful and also by questions in open format), specifically designed for this study, to measure satisfaction about the treatment program as a whole, the program components and individual experiences or recommendations, (3) interviews/evaluations with the patient advisory board and caregivers, to understand the needs and concerns of participating parents and children.

Tic severity (key outcome) was assessed by the semi-structured interview Yale Global Tic Severity Scale; YGTSS [35]. The global score (response range 0–100) is composed of an impairment score (0–50) and a total tic score (0–50). The total tic score, used as our primary outcome, adds the total motor tic score (0–25) to the total vocal tic score (0–25). We defined a 25% tic reduction as a positive response [36].

Quality of life was measured by the Gilles de la Tourette Syndrome Quality of Life Scale for children and adolescents; C&A-GTS-QOL [37], which is a 27-item disorder-specific patient-reported scale for the measurement of health-related quality of life in patients with TS (range 27–135).

Premonitory urges were assessed by the Premonitory Urges for Tics Scale; PUTS [38]. This is a nine-item self-report questionnaire which assesses tic-related feelings and sensations (premonitory urges) (range 9–36).

Emotional/behavioral functioning was measured by the Child Behavior Checklist; CBCL [39, 40]. This 113-item parent-report questionnaire assesses emotional and behavioral problems, covering 8 dimensions (raw scores were analyzed).

### Statistical analyses

To avoid chance findings, only the key outcome (tic severity) and our main secondary outcome (quality of life) were statistically tested. Other measurement outcomes were reported descriptively. To test whether there were changes in the main outcomes scores, from pre- to post-assessment to follow-up, repeated-measures ANOVAs were performed on the key outcome (tic severity) and our main secondary outcome (quality of life). The effect sizes were calculated in SPSS as Partial Eta Squared. A nonparametric test (related-samples Friedman’s two-way analyses of variance by ranks) was used as an extra control. If the parametric test and the nonparametric test lead to the same conclusion, any violations of assumptions apparently have little influence or are absent.

For each variable, complete case analysis was performed, to avoid imputation of missing data. A *p* value of < 0.05 was considered significant. To test for multiple comparisons, we did a Bonferroni correction (*p* < 0.025). SPSS Version 25 was used for the analyses (IBM SPSS Statistics for Windows, Version 25.0. Armonk, NY: IBM Corp). As a sensitivity analysis, we reanalyzed our data with including the patient who was excluded because of poor group functioning.

## Results

### Patient characteristics

A total of 27 families were interested in participating, of which 14 children were included. The 13 excluded children did not meet the criteria concerning age (4; 2 < 9 years, 2 > 17 years), tic severity (1; very mild tics), had received behavioral therapy for tics in the previous 12 months (2) or lacked motivation (2). Other reasons were: no availability left in the last group (2) and living abroad (1). However, from the eligible children who met the inclusion criteria, only one finally did not participate since the timing of the groups conflicted with school activities. Therefore, 14 patients (aged 9–14 years) diagnosed with Tourette Syndrome (TS) were included. One included patient dropped out due to poor group functioning (which was not identified during intake). A total of 13 included patients completed the full therapy program. Table 2 shows the main patient characteristics. Percentages of comorbid ASD and anxiety disorders were > 35%. Parents with a high level of education were overrepresented (57.9%).

**Table 2:** Main demographic characteristics and comorbidities, from diagnostic intake and medical files (*N* = 14)

Demographic characteristics	Group 1 (*N* = 6)	Group 2 (*N* = 8)	Total	%
Age (years), mean	12.13 (range 9.03–14.81, SD ± 1.93)	11.36 (range 9.36–13.74, SD ± 1.55)	11.69 (range 9.03–14.81, SD ± 1.70)	
Gender				
Male	4	6	10	71.4
Female	2	2	4	28.6
Cultural background				
Dutch	6	8	14	100
Parents education level				
Low (0–3)	1	0	1	5.3
Middle (4–7)	2	5	7	36.8
High (8–9)	4	7	11	57.9
Comorbidities				
ADHD	3	0	3	21.4
ASD	1	4	5	35.7
OCD	0	0	0	0
Anxiety	1	4	5	35.7
Medication for tics or comorbidities				
Aripiprazol	1	2	3	21.4
Clonidine	0	1	1	7.1
Risperidon	0	1	1	7.1
Citalopram	0	1	1	7.1
Methylphenidate	1	0	1	7.1
Haloperidol	1	0	1	7.1

*SD* standard deviation, *ADHD* attention deficit hyperactivity disorder, *ASD* autism spectrum disorder, *OCD* obsessive–compulsive disorder

### Feasibility

In the first therapy group, one boy with TS and ASD dropped out at the end of day 1 because of poor group functioning and lack of cooperation. There were no drop-outs in the second group. Both (remaining) group sizes (5 and 8) were feasible according to evaluations with the team members (behavioral therapists and patient experts). Performing the program with three therapists and eight patients offers sufficient space for an alternative program layout in parallel sessions or for extra attention in response to unexpected problems. Our team of clinicians estimated that such temporary individual moments were needed for 25% of the participating children and offered possibilities for personalizing the therapy.

Overall, parents and children reported to be satisfied about this treatment program (see appendix 1). On the treatment satisfaction questionnaires (parent and child version), the mean score was almost 4 at the five-point scale questions (mean 3.94 for children, 3.92 for parents). Children as well as parents would recommend this treatment to other families (mean score 4.77 for children, 4.23 for parents). Scores for the rating of different program components were positive regarding the ERP-sessions, psycho-education, coping strategy workshops, the ERP training app (BT-Coach) and parent meetings, and neutral on relaxation exercises. Other (open) questions asked for explanations, suggestions and remarks. Several children and parents reported the children learned a lot, had fun and felt supported. Both children and parents mentioned the time for lunchbreaks and playtime was experienced as somewhat short. Parents also reported they experienced recognition, education and support themselves. They described in what way they thought the program was helpful for their child (e.g., having a sense of control over the tics (84.6%); contact with other children with tics (38.5%)). These findings were used to evaluate the need for adaptations for future implementation of the *Tackle your Tics* program.

### Tic severity

**Fig. 1:**
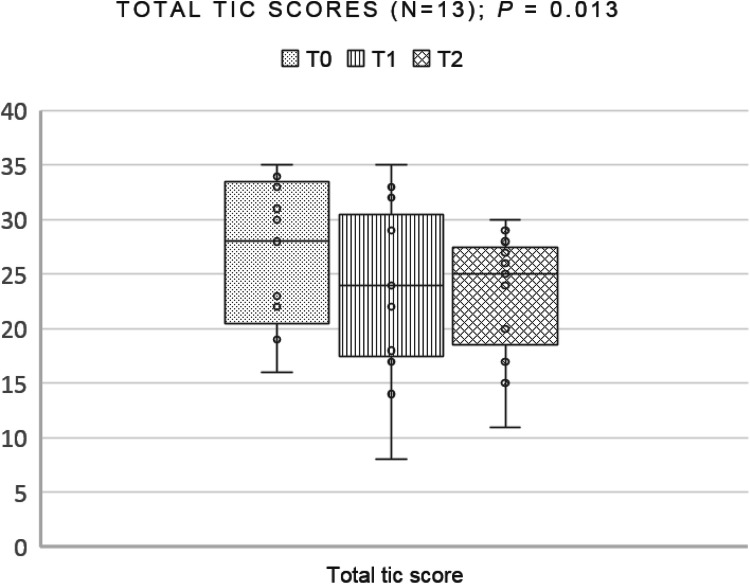
Box and whisker plot of total tic scores of treatment completers (*n* = 13) on the YGTSS at pre-treatment (T0), post-treatment (T1), and follow-up (T2). The dots show individual scores, the vertical lines (whiskers) show the ranges of scores, and the boxes show the first quartile to the third quartile with a line through the center at the median

Main treatment outcomes are shown in Table 3. The primary outcome, the mean total tic score (as measured by the YGTSS) decreased from a means of 27.08 at baseline with 4.23 points (16%) to follow-up: 3.39 points from pre- to post-treatment (23.69) and another 0.84 points to follow-up (22.85). 23.1% of the participants were rated as responders from T0 (pre-treatment) to T1 (post-treatment) and 53.8% as responders from T0 (pre-treatment) to T2 (follow-up) (data not shown in the table)]. Repeated measures analysis of variance (ANOVA) showed that the decrease on the YGTSS total tic score was significant (*p* = 0.013, effect size = 0.412). A nonparametric test (related-samples Friedman’s two-way analysis of variance by ranks), executed as an extra control test, was performed and also showed a decrease of the total tic score (*p* = 0.050). Figure 1 shows the total tic scores of treatment completers (*n* = 13). As a sensitivity analysis, we reanalyzed our data with including the complete YGTSS data (T0, T1, T2) of the patient who dropped out because of poor group functioning (n = 14). This analysis also showed a significant decrease (*p* = 0.006, effect size = 0.454, nonparametric test *p* = 0.026). Therefore, our conclusion did not depend on the inclusion of exclusion of the participant, which adds to the robustness of our inferences.

**Table 3:** Main treatment outcomes of treatment completers on T0 (pre-treatment), T1 (post-treatment) and T2 (2 months follow-up) (complete cases)

	T0				T1				T2			
	Mean	Range	95% CI	SD	Mean	Range	95% CI	SD	Mean	Range	95% CI	SD
Total tic score (YGTSS total (motoric + vocal) tic score, *n* = 13)	27.08	16–35	23.12–31.04	6.551	23.69	8–35	18.69–28.69	8.270	22.85	11–30	19.33–26.36	5.814
Motor tics (YGTSS total motor tic score, *n* = 13)	15.92	10–20	13.85–17.99	3.427	13.46	0–20	10.40–16.52	5.060	12.46	6–17	7.14–13.63	3.072
Vocal tics (YGTSS total vocal tic score, *n* = 13)	11.15	0–19	7.71–14.60	5.699	10.23	0–18	18.69–28.69	5.703	10.38	0–19	19.33–26.36	5.363
Impairment (YGTSS impairment score, *n* = 13)	24.62	10–40	18.27–30.96	10.500	14.62	0–30	7.81–21.42	11.266	16.92	0–40	9.37–24.48	12.506
Global tic severity (YGTSS global tic severity score, *n* = 13)	51.69	36–74	44.19–59.19	12.412	38.31	18–65	28.69–47.93	15.918	39.77	11–68	30.29–49.24	15.680
Quality of life (C&A-GTS-QOL total problem score, *n* = 12)	58.92	30–92	45.08–72.75	21.78	50.42	28–88	38.59–62.25	18.62	47.00	31–83	35.24–58.76	18.51
Premonitory urges (PUTS total score, *n* = 12)	21.17	15–32	18.20–24.13	4.67	20.58	9–28	16.91–24.25	5.78	20.58	11–31	16.69–24.48	6.13
Behavioral problems (CBCL total score, *n* = 9)	45.11	7–91	23.09–67.13	28.6463	39.556	5–96	16.48–62.63	30.0213	28.56	4–59	12.20–44.91	21.2786
Treatment satisfaction (children forms, *n* = 13)					3.943	3.27–4.91	3.67–4.22	0.45628				
Treatment satisfaction (parent forms, *n* = 13)					3.916	2.48–4.64	3.57–4.25	0.55926				

*95% CI* 95% confidence interval for mean, *YGTSS* Yale Global Tic Severity Scale, *PUTS* Premonitory Urge for Tics Scale, *C&A-GTS-QOL* The Gilles De La Tourette Syndrome-Quality of Life Scale for Children and Adolescents, *CBCL* Children Behavior Checklist

The mean score for motor tics decreased with 3.46 points (22%) from baseline to follow-up. Vocal tics decreased with 0.77 points (7%) from baseline to follow-up. Functional impairment (as measured by the YGTSS) decreased with 7.7 points (31%) from baseline to follow-up.

### Quality of life

Overall, the scores of this sample on a quality of life questionnaire (C&A-GTS-QOL total problem score) improved significantly, with 14% from pre- to post-treatment and 20% from pre-treatment to follow-up (*p* = 0.002, using repeated measures analysis of variance, ANOVA, effect size = 0.584). A nonparametric test (related-samples Friedman’s two-way analysis of variance by ranks), performed as an extra control test, also showed a significant decrease of the total problem score (*p* = 0.002). As a sensitivity analysis, we reanalyzed our data for quality of life with including the patient who dropped out because of poor group functioning (*n* = 13), imputing missing data from T1 and T2 for this patient (based on the baseline measurement on T0). This analysis also showed a significant decrease (*p* = 0.003, effect size = 0.539, nonparametric test *p* = 0.002). Therefore, our conclusion did not depend on the inclusion of exclusion of the participant, which adds to the robustness of our inferences.

### Other secondary outcomes

Descriptively statistic showed premonitory urges scores decreased with 3%, which is in line with previous studies in which premonitory urges showed no difference from pre- to post-treatment (e.g., [41]). Behavioral and emotional problems, as measured by the Child Behavioral Checklist (CBCL) decreased with 37% between pre-treatment and follow-up. Due to technical (online) problems, data on behavioral/emotional problems were complete for only nine children.

### Group, gender and age differences

As can be seen in appendix 2, children in the second group, girls and older children showed more improvement.

## Discussion

This study shows promising results regarding the feasibility of intensive ERP therapy as offered in the *Tackle your Tics* program. In line with our hypotheses, the treatment program was feasible and satisfactory for participating children and their families. The results on treatment outcome show indications of effectiveness of the program, to improve both tic severity and quality of life.

Despite these promising improvements on tic severity and quality of life, mean total tic scores decreased only 16% from pre-treatment to follow-up in this study. Impairment scores improved 41% pre- to post-treatment (31% pre-treatment to follow-up). In previous studies on behavioral therapy for tics, these percentages were higher. In a meta-analysis by McGuire et al. [16], YGTSS total tic scores decreased around 30% in most studies. A study of an individual, weekly treatment program ‘Living With Tics’ [7], aimed to reduce impairment and improve quality of life, found a total tic reduction of 30% and a reduction of 70% in impairment score (as measured by the YGTSS).

Improvement of quality of life (13% measured by the PedsQL, Pediatric Quality of Life Inventory) was comparable with the results of our *Tackle your Tics* study (14% pre- to post-treatment, 20% to follow-up).

There are several explanations for these findings. First, Blount et al. [20] suggested that patients who receive intensive treatment may not have the opportunity to practice their newly acquired skills within their normal environment until after treatment is complete. Possibly, children in our *Tackle your Tics* study did improve quickly but did fewer exercises during and after the treatment period. Since post-treatment measurements were performed a week after the last treatment day (to be able to measure the last week’s tic severity by the YGTSS), these improvements may not (yet) have been generalized to the home environment. We expected that children and parents would have more time and motivation to practice at home between post-treatment measurement and follow-up. At follow-up, only seven children (54%) reported that they continued doing ERP exercises after the treatment days on a regular basis. Of these children, 6 were responders with over 25% total tic reduction from pre-treatment to follow-up. The low rate of children who reported to have practiced at home can be explained by the following possibilities: (1) parents did receive education about the therapy but no hands-on training; therefore, parents may not have helped their children sufficiently with the exercises; (2) children learned to accept their tics in the coping strategy workshops, which may have reduced the need to exercise and reduce their tics. In a get together meeting with participating parents and children (after completion of all treatments and measurements), several parents reported their children felt no necessity or obligation towards a therapist anymore, after the treatment was finished. Based on this feedback and the findings from the treatment satisfaction questionnaire, we recommend to add the following elements in future use of the *Tackle your Tics* program: (1) parent involvement during therapy sessions, to train parents how to help their children during home exercises, and (2) an extra booster afternoon after 1 month, to keep the children motivated to practice at home. Finally, some parents and children stated they now felt more in control of tics and did not feel the necessity to continue exercising all the time, since they now know how to reduce tics in time of stress and when they wanted to.

However, previous studies [16, 22] found that intensive individual treatment lead to improvement comparable to care as usual (weekly individual therapy sessions). In studies on intensive behavioral therapies for children with anxiety disorders or OCD, it has been suggested that condensing the spacing of sessions can optimize conditions for reducing symptoms, by maximizing (extinction) learning by massed practice, increased family’s attention on treatment, faster reduction of functional impairment and better therapists’ monitoring of adherence [24, 26, 43]. The intensive format also facilitates patients to dedicate time specifically to detecting urges and tics and implement their treatment exercises without the distractions of day-to-day life [44]. Our clinical impression of the team was that progress in therapy sessions seemed to be achieved very quickly in both groups, although not formally measured with (intermediate) measurements during therapy. After the first day, several disturbing tics (e.g. eye rolling, face rubbing) were already tackled in some children. On day 3, children went outside of the treatment center (e.g., riding a bike, being among other people, playing games) with the therapists to learn to generalize their newly learned skills. The small decrease in vocal tics (7%) may be explained by the fact that therapists focused on the tics that were most present and distressing to the child. These tics were mostly motoric tics that impaired concentration and activities or were painful.

Second, the group setting may have influenced the treatment outcome. The therapy sessions were partly in small groups of 2 or 3 children, who may have had less individual practice while supporting other children. Nissen et al. [41] compared behavioral treatments for tics (habit reversal training and ERP) in a group setting to individual training, and found no significant differences in total tic scores, but individual training showed significantly greater reduction in the functional impairment score and negative thoughts and interpretations of their tics. They suggest that in an individual setting, the interaction between therapist and the child is more direct, and the therapist is able to focus more intensely on a particular child’s resources and difficulties. Also, group processes may have influenced the treatment outcome. As can be seen in Table 2, the children with comorbid ASD and anxiety disorders were mainly in the same group (group 2, *N* = 8). Our clinical impression was that this group provided a safe and quiet environment, in which children may have benefited more from the program than children in the first group (group 1, *N* = 6). In group 1, ADHD symptoms were more prominent (50%) and the group—although smaller—was described by the therapists and patient representatives as more restless (although group dynamics were also positive and supportive). Appendix 2 shows that treatment outcomes of group 2 improved more, which may be an indication of difference in the group participants. However, previous studies [27, 41, 45] suggest that group therapy for children with tics appeared feasible and has comparable treatment outcomes as individual therapy. Being around other children with tics did not increase tic expression. Moreover, benefits of group-based treatment studies have been reported: reducing waiting lists, increasing the cost-effectiveness and providing peer support and a safe space for sharing experiences (S. Zimmerman et al. Comprehensive Behavioral Intervention for Tics vs Psychoeducational-Supportive treatments in group setting for children with chronic tic disorders: A randomized controlled trial. Oral presentation at the European Conference on Tourette Syndrome and Tic Disorders 2018).

Both parents and children reported a wide range of scores (1–5) in the treatment satisfaction forms on the question “Have your/your child’s tics been reduced?” at post-treatment. One parent and one child did not experience any reduction. Interestingly, parents and children responded positively on the question: “How helpful was this therapy week for you/your child’s problems?” Most of them mentioned that having a sense of control over the tics and to be able to suppress them temporarily, was helpful (84.6%) which was also found in the study of Nissen et al. [41] in group settings as well as individual settings. Also, many parents reported that the contact with other children with tics and being not the only one with tics, was helpful (38.5%).

This study is, to our knowledge, the first study worldwide into a brief, intensive group-based exposure therapy for children with chronic tic disorders. Considering the promising outcomes, we recommend to study the effectiveness in larger trials. If this type of treatment is shown effective, this program can offer several benefits. It can expand the access to behavioral treatment for children and adults living in different areas, without local specialized therapist trained in treatment of tics. Families find it easier to travel for a consecutive series of days (or possibly stay overnight) than to travel for a longer period for weekly individual sessions. Furthermore, an advantage is that brief intensive therapy can bring improvements in daily functioning in a much shorter time period, so that children can benefit earlier. In this shorter period of time, different supporting components can be offered simultaneously, among which coping strategy workshops and parent meetings organized by the patient organization. The treatment program offers opportunities to educate behavioral therapists in exposure and response prevention therapy for tics. When future research shows which children benefit most from this intensive group-based format and for which children individual care as usual in 12 weekly sessions is recommended, this knowledge will offer opportunities to personalize treatment advice in the future.

### Strengths and limitations

Although our results are promising, we have to keep in mind several limitations of this study. The sample size was small (*N* = 14) with no control group for the natural course of tics and other symptoms. An alternative would have been to assess a baseline phase and to compare the baseline phase with the treatment phase (e.g., Viefhaus et al. [46]). Since there was only one treatment condition, the YGTSS interview could not be rated blinded. No objective measurement of tic severity like video rating was used. Furthermore, the education level of the parents seems to be unrepresentative high may have had a positive influence on the treatment outcomes.

Therefore, no firm conclusions about effectiveness of the *Tackle your Tics* program on tics and other symptoms can be drawn. The results of the ANOVAs and the non parametrical tests showed same results on the outcomes tic severity and quality of life (which can be considered as strengthening our conclusions), with one exception: after Bonferroni correction, the results on tic severity, with non parametrical testing, became non significant (*p* = 0.050). However, the Bonferroni correction can be considered as too conservative. Future studies with larger sample sizes, using a control group are needed.

## Conclusion

The present study provides first insight in possibilities to offer a brief, intensive and comprehensive therapy group program. It suggests that *Tackle your Tics* is a feasible and promising program for children with chronic tic disorders, to improve both tic severity as well as quality of life.

## Appendix 1

See Table

**Table 4:** Treatment satisfaction scores on a 1–5 scale questionnaire (*N* = 13)

	Mean	Range	SD
Children questionnaire			
General questions (1–5 points scale):			
Q1: What did you think of the number of days?*	4.38	3–5	0.961
Q2: Was the training as you expected?	3.92	3–5	0.760
Q3: Did you understand everything well?	4.31	3–5	0.751
Q5: Have your tics been reduced?	3.08	1–5	1.038
Q10: What rating would you give the entire week?*	4.31	3–5	0.630
Q11: Would you recommend this therapy week to other children with tics?	4.77	4–5	0.515
Parents questionnaire			
General questions (1–5 points scale):			
Q1: How helpful was this week for your child?	4.23	2–5	1.013
Q15: Was the therapy and the explanation you received about it well understood?	4.31	4–5	0.480
Q16: Have you received enough tools to help your child practice, even after the therapy?	3.77	3–4	0.439
Q21: Did this message (tics can be controlled but are not wrong) come across sufficiently?	3.85	1–5	0.987
Q23: What did you think of the extent to which you were involved as a parent in the therapy week?*	4.15	2–5	1.345
Q24: Are you satisfied with this form of treatment?	4.15	2–5	0.899
Q25: Would you recommend this intensive form to other parents of children with tics?	4.23	2–5	0.832

This table shows the answers on the general 5-point Likert scale questions (1 = very negative/not helpful at all; 5 = very positive/helpful)

*Questions with other response categories that have been converted to a 5-point scale

4.

## Appendix 2

See Table

**Table 5:** Supplemental data: group, gender and age differences

Group	T0		T1		T2	
	Group 1 (*N* = 6)	Group 2 (*N* = 8)	Group 1	Group 2	Group 1	Group 2
Tic severity (mean YGTSS total tic score, *n* = 13)	28.00	27.25	24.83	22.75	23.67	22.25
Quality of life (mean C&A-GTS-QOL total problem score, *n* = 12)	58.50	56.88	58.50	46.38	55.00	42.38
Gender	Male (*N* = 10)	Female (*N* = 4)	Male	Female	Male	Female
Tic severity (mean YGTSS total tic score, *n* = 13)	25.78	30.00	23.78	23.50	22.11	24.50
Quality of life (mean C&A-GTS-QOL total problem score, *n* = 12)	55.22	66.50	50.75	49.75	46.44	49.00
Age	9–11 (*N* = 9)	12–14 (*N* = 5)	9–11	12–14	9–11	12–14
Tic severity (mean YGTSS total tic score, *n* = 13)	25.33	31.00	24.33	22.25	22.67	23.25
Quality of life (mean C&A-GTS-QOL total problem score, *n* = 12)	55.78	65.25	50.63	50.00	46.44	49.00

*YGTSS* Yale Global Tic Severity Scale, *C&A-GTS-QOL* The Gilles De La Tourette Syndrome-Quality of Life Scale for Children and Adolescents

5.

## References

[1] Hirschtritt ME, Lee PC, Pauls DL (2015). Lifetime prevalence, age of risk, and genetic relationships of comorbid psychiatric disorders in tourette syndrome. JAMA Psychiatry.

[2] Knight T, Steeves T, Day L (2012). Prevalence of tic disorders: a systematic review and meta-analysis. Pediatr Neurol.

[3] Robertson MM (2008). The prevalence and epidemiology of Gilles de la Tourette syndrome: part 1: the epidemiological and prevalence studies. J Psychosom Res.

[4] Conelea CA, Woods DW, Zinner SH (2013). The impact of Tourette syndrome in adults: results from the Tourette syndrome impact survey. Community Ment Health J.

[5] Storch EA, Lack CW, Simons LE (2007). A measure of functional impairment in youth with Tourette’s syndrome. J Pediatr Psychol.

[6] Cutler D, Murphy T, Gilmour J, Heyman I (2009). The quality of life of young people with Tourette syndrome. Child Care Health Dev.

[7] McGuire JF, Arnold E, Park JM (2015). Living with tics: reduced impairment and improved quality of life for youth with chronic tic disorders. Psychiatry Res.

[8] Kraft JT, Dalsgaard S, Obel C (2012). Prevalence and clinical correlates of tic disorders in a community sample of school-age children. Eur Child Adolesc Psychiatry.

[9] McGuire JF, Hanks C, Lewin AB (2013). Social deficits in children with chronic tic disorders: phenomenology, clinical correlates and quality of life. Compr Psychiatry.

[10] Pérez-Vigil A, De La Cruz LF, Brander G (2018). Association of Tourette syndrome and chronic tic disorders with objective indicators of educational attainment: a population-based sibling comparison study. JAMA Neurol.

[11] Cath DC, Hedderly T, Ludolph AG (2011). European clinical guidelines for Tourette syndrome and other tic disorders. Part I: assessment. Eur Child Adolesc Psychiatry.

[12] Fernández de la Cruz L, Rydell M, Runeson B (2017). Suicide in Tourette’s and chronic tic disorders. Biol Psychiatry.

[13] Eddy CM, Cavanna AE, Gulisano M (2011). Clinical correlates of quality of life in Tourette syndrome. Mov Disord.

[14] European Tourette Syndrome Research Survey 2017–18. https://www.tourettes-action.org.uk/news-294-european-tourette-syndrome-research-survey-2017-18.html. Accessed 28 Jul 2019

[15] Verdellen C, van de Griendt J, Hartmann A, Murphy T (2011). European clinical guidelines for Tourette Syndrome and other tic disorders. Part III: behavioural and psychosocial interventions. Eur Child Adolesc Psychiatry.

[16] McGuire JF, Piacentini J, Brennan EA (2014). A meta-analysis of behavior therapy for Tourette Syndrome. J Psychiatr Res.

[17] Verdellen CWJ, Keijsers GPJ, Cath DC, Hoogduin CAL (2004). Exposure with response prevention versus habit reversal in Tourettes’s syndrome: a controlled study. Behav Res Ther.

[18] Van de Griendt JMTM, Verdellen CWJ, van Dijk MK, Verbraak MJPM (2013). Behavioural treatment of tics: habit reversal and exposure with response prevention. Neurosci Biobehav Rev.

[19] Woods DW, Conelea CA, Walther MR (2007). Barriers to dissemination: exploring the criticisms of behavior therapy for tics. Clin Psychol Sci Pract.

[20] Blount TH, Raj JJ, Peterson AL (2018). Intensive outpatient comprehensive behavioral intervention for tics: a clinical replication series. Cogn Behav Pract.

[21] Taylor C, Greenhalgh J, Stark D (2017). C2.1 Delivery of behavioural interventions for tics in an intensive outpatient format followed by remote delivery: a UK paediatric case series. Arch Dis Child.

[22] Storch EA, Geffken GR, Merlo LJ (2007). Family-based cognitive-behavioral therapy for pediatric obsessive-compulsive disorder. J Am Acad Child Adolesc Psychiatry.

[23] Whiteside SP, Jacobsen AB (2010). An uncontrolled examination of a 5-day intensive treatment for pediatric OCD. Behav Ther.

[24] Whiteside SPH, Dammann JE, Tiede MS (2018). Increasing availability of exposure therapy through intensive group treatment for childhood anxiety and OCD. Behav Modif.

[25] Hendriks L, de Kleine RA, Heyvaert M (2017). Intensive prolonged exposure treatment for adolescent complex posttraumatic stress disorder: a single-trial design. J Child Psychol Psychiatry Allied Discip.

[26] Öst L-G, Ollendick TH (2017). Brief, intensive and concentrated cognitive behavioral treatments for anxiety disorders in children: a systematic review and meta-analysis. Behav Res Ther.

[27] Yates R, Edwards K, King J (2016). Habit reversal training and educational group treatments for children with tourette syndrome: a preliminary randomised controlled trial. Behav Res Ther.

[28] Dabrowski J, King J, Edwards K, et al (2017) 81 Group interventions for children with tourette syndrome: a 12 month follow up study of a randomised controlled trial comparing comprehensive behavioural intervention and psycho-education. In: Poster presentation abstracts. BMJ Publishing Group Ltd and Royal College of Paediatrics and Child Health, p A25.3–A26

[29] Himle JA, Fischer DJ, Van Etten ML (2003). Group behavioral therapy for adolescents with tic-related and non-tic-related obsessive-compulsive disorder. Depress Anxiety.

[30] American Psychiatric Association (2013). DSM-V diagnostic and statistical manual of mental disorders.

[31] Verdellen C, Van de Griendt J, Kriens S, Van Oostrum I (2011). Tics—therapist manual and workbook for children.

[32] Hoogduin K, Verdellen C, Cath D (1997). Exposure and response prevention in the treatment of Gilles de la Tourette’s syndrome: four case studies. Clin Psychol Psychother.

[33] van de Griendt J, Verdellen C, Van Liempt T (2016) BT-Coach—behaviour therapy for tics and Tourette Syndrome. https://www.bt-tics.com/bt-coach. Accessed 30 Oct 2019

[34] Silverman WK, Alban AM (2001). ADIS-C | anxiety disorders interview schedule.

[35] Leckman JF, Riddle MA, Hardin MT (1989). The Yale Global Tic Severity Scale: initial testing of a clinician-rated scale of tic severity. J Am Acad Child Adolesc Psychiatry.

[36] Jeon S, Walkup JT, Woods DW (2013). Detecting a clinically meaningful change in tic severity in Tourette syndrome: a comparison of three methods. Contemp Clin Trials.

[37] Cavanna AE, Luoni C, Selvini C (2013). The Gilles de la Tourette Syndrome-Quality of Life Scale for children and adolescents (C&A-GTS-QOL): development and validation of the Italian version. Behav Neurol.

[38] Woods DW, Piacentini J, Himle MB, Chang S (2005). Premonitory Urge for Tics Scale (PUTS): Initial psychometric results and examination of the premonitory urge phenomenon in youths with tic disorders. J Dev Behav Pediatr.

[39] Achenbach T, Edlebrock C (1993). Manual for the child behavior checklist and revised child behavior profile.

[40] Verhulst FC, Van der Ende J (2013). Handleiding ASEBA-Vragenlijsten voor leeftijden 6 t/m 18 jaar: CBCL/6-18.

[41] Nissen JB, Kaergaard M, Laursen L (2019). Combined habit reversal training and exposure response prevention in a group setting compared to individual training: a randomized controlled clinical trial. Eur Child Adolesc Psychiatry.

[42] Capriotti MR, Woods DW (2013). Cognitive-behavioral treatment for tics. Tourette syndrome.

[43] Craske MG, Treanor M, Conway CC (2014). Maximizing exposure therapy: an inhibitory learning approach. Behav Res Ther.

[44] Blount TH, Lockhart A-LT, Garcia RV (2014). Intensive outpatient comprehensive behavioral intervention for tics: a case series. World J Clin cases.

[45] Dabrowski J, King J, Edwards K (2018). The long-term effects of group-based psychological interventions for children with tourette syndrome: a randomized controlled trial. Behav Ther.

[46] Viefhaus P, Feldhausen M, Görtz-Dorten A (2019). A new treatment for children with chronic tic disorders—resource activation. Psychiatry Res.

